# Supplementary material for: Integrating systemic and molecular levels to infer key drivers sustaining metabolic adaptations
Source: PLoS Comput Biol. 2021 Jul 23;17(7):e1009234. doi: 10.1371/journal.pcbi.1009234 (PMC8336858; doi:10.1371/journal.pcbi.1009234)
Supplement: S1 Table — (PDF) [file pcbi.1009234.s001.pdf]

S1 Table. List of metabolites.

ID (x_i)		description
x_{01}	ACoA.c	cytoplasmic acetyl-CoA
x_{02}	ACoA.m	mitochondrial acetyl-CoA
x_{03}	ADP	ADP
x_{04}	aKG	α-ketoglutarate
x_{05}	Ala	alanine
x_{06}	Arg	arginine
x_{07}	Asn	asparagine
x_{08}	Asp	aspartate
x_{09}	ATP	ATP
x_{10}	c3PG	3-phosphoglycerate
x_{11}	Cit	citrate
x_{12}	CoA.c	cytoplasmic coenzyme A
x_{13}	CoA.m	mitochondrial coenzyme A
x_{14}	Cys	cysteine
x_{15}	DHAP	dihydroxyacetone phosphate
x_{16}	E4P	erythrose 4-phosphate
x_{17}	F6P	fructose 6-phosphate
x_{18}	FBP	fructose 1,6-bisphosphate
x_{19}	Fum	fumarate
x_{20}	G3P	glyceraldehyde 3-phosphate
x_{21}	G6P	glucose 6-phosphate
x_{22}	Glc	glucose
x_{23}	Gln	glutamine
x_{24}	Glu	glutamate
x_{25}	Gly	glycine
x_{26}	His	histidine
x_{27}	Ile	isoleucine

ID (x_i)	ID	description
x_{28}	Lac	lactate
x_{29}	Leu	leucine
x_{30}	Lys	lysine
x_{31}	Mal	malate
x_{32}	Met	methionine
x_{33}	NADH.c	cytoplasmic NADH
x_{34}	NADH.m	mitochondrial NADH
x_{35}	NADP	cytoplasmic NADP
x_{36}	NADPH	cytoplasmic NADPH
x_{37}	NAD.c	cytoplasmic NAD
x_{38}	NAD.m	mitochondrial NAD
x_{39}	OAA.c	cytoplasmic oxaloacetate
x_{40}	OAA.m	mitochondrial oxaloacetate
x_{41}	P5C	Δ1-pyrroline-5-carboxylate
x_{42}	PenP	PenP, pentose phosphate pool (ribulose 5-phospate, ribose 5-phosphate, xylulose 5-phosphate)
x_{43}	PEP	phosphoenol pyruvate
x_{44}	Phe	phenylalanine
x_{45}	Pro	proline
x_{46}	Pyr	pyruvate
x_{47}	S7P	sedoheptulose 7-phosphate
x_{48}	Ser	serine
x_{49}	Suc	succinate
x_{50}	Thr	threonine
x_{51}	Trp	tryptophan
x_{52}	Tyr	tyrosine
x_{53}	Val	valine

Name.c for only-cytoplasmic compounds, *name.m* for only-mitochondrial compounds.
