# Supplementary material for: Integrating systemic and molecular levels to infer key drivers sustaining metabolic adaptations
Source: PLoS Comput Biol. 2021 Jul 23;17(7):e1009234. doi: 10.1371/journal.pcbi.1009234 (PMC8336858; doi:10.1371/journal.pcbi.1009234)
Supplement: S2 Table — (PDF) [file pcbi.1009234.s002.pdf]

S2 Table. List of reactions, stoichiometry, reaction fluxes, and disequilibrium ratios.

ID	Substrates	Products	net Flux (J_j) (pmol·cell^{-1}·h^{-1})	$\rho = \frac{vr}{vf}$	Description
R_{01}		Glc	0.48235	0.90	Glucose transport (uptake) (Glc transp)
R_{02}	ATP + Glc	ADP + G6P	0.48235	0.00	Hexokinase (HK)
R_{03}	G6P	F6P	0.43337	0.90	Glucose-6-phosphate isomerase (Ala transp)
R_{04}	ATP + F6P	ADP + FBP	0.46542	0.00	Phosphofructokinase (PFK)
R_{05}	FBP	DHAP + G3P	0.46542	0.75	Aldolase
R_{06}	DHAP	G3P	0.46542	0.90	Triosephosphate isomerase
R_{07}	ADP + G3P + NAD.c	ATP + c3PG + NADH.c	0.94687	0.90	Glyceraldehyde 3-phosphate dehydrogenase & phosphoglycerate kinase
R_{08}	c3PG	PEP	0.94353	0.90	Phosphoglycerate mutase & enolase (PGM/ENO)
R_{09}	ADP + PEP	ATP + Pyr	0.94353	0.00	Pyruvate kinase (PK)
R_{10}	NADH.c + Pyr	Lac + NAD.c	0.89943	0.06	Lactate dehydrogenase
R_{11}	Lac		0.89943	0.90	Lactate transport (release)
R_{12}	G6P + 2*NADP	2*NADPH + PenP	0.04808	0.00	Glucose 6-phosphate dehydrogenase & 6-phosphogluconolactonase & phosphogluconate dehydrogenase & ribose-5-phosphate isomerase & ribulose-phosphate 3-epimerase (oxidative pentose-phosphate pathway; oxid. PPP)
R_{13}	E4P + PenP	F6P + G3P	0.01603	0.00	Transketolase (TKT-1)
R_{14}	2*PenP	G3P + S7P	0.01603	0.76	Transketolase (TKT-2)
R_{15}	G3P + S7P	E4P + F6P	0.01603	0.90	Transaldolase (TA)
R_{16}		Ser	0.01802	0.90	Serine transport (uptake)
R_{17}	c3PG + Glu + NAD.c	aKG + NADH.c + Ser	0.00328	0.00	Phosphoglycerate dehydrogenase & phosphoserine transaminase & phosphoserine phosphatase
R_{18}	Ser	Pyr	0.00808	0.00	Serine dehydratase & spontaneous reactions to pyruvate
R_{19}	CoA.m + NAD.m + Pyr	ACoA.m + NADH.m	0.08405	0.00	Pyruvate dehydrogenase complex (PDH)
R_{20}	ACoA.m + OAA.m	Cit + CoA.m	0.12063	0.00	Citrate synthase
R_{21}	Cit + NADP (NAD.m)	aKG + NADPH (NADH.m)	0.10143	0.00	Isocitrate dehydrogenase & aconitase (ACO/IDH) (mitochondrial and cytosolic activities)
R_{22}	ADP + aKG + NAD.m	ATP + NADH.m + Suc	0.15123	0.00	α-ketoglutarate dehydrogenase & succinyl-CoA synthetase (αKGDH/SCS)
R_{23}	1.5*ADP + Suc	1.5*ATP + Fum	0.15798	0.00	Succinate dehydrogenase; oxidative phosphorylation from complex II of respiratory chain (SDH/CII)
R_{24}	2.5*ADP + NADH.m	2.5*ATP + NAD.m	0.43913	0.00	NADH dehydrogenase; oxidative phosphorilation from complex I of respiratory chain
R_{25}	Fum	Mal	0.15843	0.90	Fumarate hydratase (FH)
R_{26}	Mal + NAD.m	NADH.m + OAA.m	0.15283	0.00	Malate dehydrogenase (MDH.m) (mitochondrial)
R_{27}	ATP + Pyr	ADP + OAA.m	0.00787	0.00	Pyruvate carboxylase (PC)
R_{28}	ATP + Cit + CoA.c	ACoA.c + ADP + OAA.c	0.01925	0.00	Citrate lyase (ACLY)
R_{29}	NADH.c + OAA.c	Mal + NAD.c	0.05072	0.90	Malate dehydrogenase (MDH.c) (cytosolic)
R_{30}	Mal + NADP (NAD.m)	NADPH (NADH.m) + Pyr	0.05632	0.00	Malic enzyme (mitochondrial and cytosolic activities) (ME)
R_{31}	Glu + Pyr	aKG + Ala	0.02010	0.15	Alanine transaminase (ALT)
R_{32}	Ala		0.01452	0.90	Alanine transport (release)
R_{33}	Glu + OAA.m	aKG + Asp	0.04012	0.00	Aspartate transaminase (ASP)
R_{34}	aKG + Asp	Glu + OAA.c	0.03147	0.90	Aspartate transaminase (ASP)

R_{35}		Gln	0.06613	0.90	Glutamine transport (uptake) (Gln transp)
R_{36}	Gln	Glu	0.06310	0.14	Glutaminase (GLS) (and Glutamine synthase)
R_{37}	Glu + NAD.m (NADP)	aKG + NADH.m (NADPH)	0.03430	0.00	Glutamate dehydrogenase (GDH)
R_{38}	Glu		0.01057	0.00	Glutamate transport (release) (Glu transp)
R_{39}		Arg	0.00807	0.00	Arginine transport (uptake)
R_{40}	Asn		0.00136	0.00	Asparagine transport (release)
R_{41}	Asp		0.00122	0.00	Aspartate transport (release)
R_{42}		Cys	0.00372	0.00	Cysteine transport (uptake)
R_{43}	Gly		0.00298	0.00	Glycine transport (release)
R_{44}		His	0.00167	0.00	Histidine transport (uptake) (His transp)
R_{45}		Ile	0.00600	0.00	Isoleucine transport (uptake)
R_{46}		Leu	0.00712	0.00	Leucine transport (uptake)
R_{47}		Lys	0.00637	0.00	Lysine transport (uptake)
R_{48}		Met	0.00247	0.00	Methionine transport (uptake)
R_{49}		Phe	0.00250	0.00	Phenylalanine transport (uptake)
R_{50}	Pro		0.00100	0.90	Proline transport (release)
R_{51}		Thr	0.00508	0.00	Threonine transport (uptake)
R_{52}		Trp	0.00042	0.00	Tryptophan transport (uptake) (Trp transp)
R_{53}		Tyr	0.00168	0.00	Tyrosine transport (uptake)
R_{54}		Val	0.00510	0.00	Valine transport (uptake)
R_{55}	ATP + G6P	ADP	0.00090	0.00	Phosphoglucomutase & glucose incorporation for glycogen synthesis
R_{56}	0.088*Ala + 0.055*Arg + 0.042*Asn + 0.053*Asp + 4.3*ATP + 0.021*Cys + 0.047*Gln + 0.057*Glu + 0.079*Gly + 0.021*His + 0.048*Ile + 0.083*Leu + 0.084*Lys + 0.02*Met + 0.032*Phe + 0.046*Pro + 0.063*Ser + 0.057*Thr + 0.006*Trp + 0.027*Tyr + 0.061*Val	4.3*ADP	0.06380	0.00	Amino acid incorporation for protein synthesis
R_{57}	8*ACoA.c + 7*ATP + 14*NADPH	7*ADP + 8*CoA.c + 14*NADP	0.00240	0.00	Synthesis of palmitate
R_{58}	Ser	Gly	0.00802	0.89	Serine hydroxymethyltransferase
R_{59}	aKG + Arg	Glu + P5C	0.00453	0.00	Arginase & ornithine aminotransferase
R_{60}	NADPH + P5C	NADP + Pro	0.00393	0.00	Pyrroline-5-carboxylate reductase (PYCR)

R_{61}	NAD.m + P5C	Glu + NADH.m	0.00063	0.90	Overall reaction involved in the synthesis (glutamate-5-semialdehyde dehydrogenase & spontaneous equilibrium P5C-GSA) and degradation (1-pyrroline-5-carboxylate dehydrogenase) of proline and arginine
R_{62}	aKG + CoA.m + Ile + 2*NAD.m	ACoA.m + Glu + 2*NADH.m + Suc	0.00292	0.00	Overall reaction for isoleucine degradation: 3-methyl-2-oxopentanoate dehydrogenase (branched-chain α-keto acid dehydrogenase complex) & other 9 steps via 3-methyl-2-oxopentanoate, propanoyl-CoA and succinyl-CoA
R_{63}	aKG + ATP + 3*CoA.m + Leu + NAD.m	3*ACoA.m + ADP + Glu + NADH.m	0.00183	0.00	Overall reaction for leucine degradation: 4-methyl-2-oxopentanoate dehydrogenase (branched-chain α-keto acid dehydrogenase complex) & other 7 steps via 4-methyl-2-oxopentanoate, isovaleryl-CoA and acetoacetate
R_{64}	2*aKG + 2*CoA.m + Lys + 2*NAD.m	2*ACoA.m + 2*Glu + 2*NADH.m	0.00100	0.00	Overall reaction for lysine degradation: lysine-α-ketoglutarate reductase & other 9 steps via saccharopine, glutaryl-CoA and acetoacetyl-CoA
R_{65}	Met + NAD.m + Ser	Cys + NADH.m + Suc	0.00122	0.00	Overall reaction for methionine degradation: methionine adenosyltransferase & other 11 steps via S-adenosyl-L-methionine, homocysteine, cystathionine, 2-oxobutanoate, propanoyl-CoA, and succinyl-CoA
R_{66}	NAD.m + Thr	NADH.m + Suc	0.00146	0.00	Overall reaction for threonine degradation: threonine deaminase (serine dehydratase) & 2-oxobutanoate dehydrogenase & propionyl-CoA carboxylase & methylmalonyl-CoA epimerase & methylmalonyl-CoA mutase & succinyl-CoA ligase
R_{67}	2*CoA.m + 2*NAD.m + Trp	2*ACoA.m + Ala + 2*NADH.m	0.00004	0.00	Overall reaction for tryptophan degradation: tryptophan 2,3-dioxygenase & other 14 steps via synthesis of kynurenine, glutaryl-CoA and acetoacetyl-CoA
R_{68}	aKG + 3*NAD.m + Val	Glu + 3*NADH.m + Suc	0.00120	0.00	Overall reaction for valine degradation: 3-methyl-2-oxobutanoate dehydrogenase (branched-chain α-keto acid dehydrogenase complex) & other 10 steps via 3-methyl-2-oxobutanoate, propanoyl-CoA and succinyl-CoA
R_{69}	Asp	Asn	0.00403	0.90	Overall reaction covering the synthesis (asparagine synthetase) and degradation (asparaginase) of asparagine
R_{70}	aKG + Cys	Glu + Pyr	0.00357	0.00	Overall reaction for cysteine degradation: cysteine dioxygenase & 3-sulfino-alanine aminotransferase |cysteine aminotransferase & 3-mercaptopyruvate sulfurtransferase
R_{71}	His	Glu	0.00029	0.00	Overall reaction for histidine degradation: histidase & imidazole propionase & glutamate formimidoyltransferase
R_{72}	Phe	Tyr	0.00045	0.00	Phenylalanine hydroxylase
R_{73}	aKG + Tyr	Fum + Glu	0.00041	0.00	Overall reaction for tyrosine degradation: tyrosine aminotransferase & 4-hydroxyphenylpyruvate dioxygenase & homogentisate 1,2-dioxygenase & maleylacetoacetate isomerase & fumarylacetoacetate hydrolase
R_{74}	ATP	ADP	2.10763	0.00	Additional ATP consumption
R_{75}	NADPH	NADP	0.21633	0.00	Additional NADPH utilization
R_{76}	CoA.m	ACoA.m	0.02610	0.00	Mitochondrial acetyl-CoA recycling

Flux values are the average values of the best fits for control cells and cells with CDK4/6 inhibited.
