# Supplementary material for: Integrating systemic and molecular levels to infer key drivers sustaining metabolic adaptations
Source: PLoS Comput Biol. 2021 Jul 23;17(7):e1009234. doi: 10.1371/journal.pcbi.1009234 (PMC8336858; doi:10.1371/journal.pcbi.1009234)
Supplement: S3 Table — (PDF) [file pcbi.1009234.s003.pdf]

S3 Table. List of initial domains with reduced bounds.

ID	Measured changes in fluxes: $\Delta\log_2 J$	
	minimum	maximum
R_{01}	-0.24	-0.21
R_{02}	-0.24	-0.21
R_{03}	-0.27	-0.19
R_{04}	-0.24	-0.21
R_{05}	-0.24	-0.21
R_{06}	-0.24	-0.21
R_{07}	-0.24	-0.21
R_{08}	-0.24	-0.21
R_{09}	-0.24	-0.21
R_{10}	-0.18	-0.15
R_{11}	-0.18	-0.15
R_{12}	-0.66	0.12
R_{13}	-0.66	0.12
R_{14}	-0.66	0.12
R_{15}	-0.66	0.12
R_{16}	-0.18	0.01
R_{17}	-0.67	0.22
R_{18}	-0.34	0.27
R_{19}	-0.73	-0.58
R_{20}	-0.57	-0.48
R_{21}	-0.18	-0.02
R_{22}	-0.05	0.00
R_{23}	-0.03	0.02
R_{24}	-0.25	-0.20
R_{25}	-0.03	0.02
R_{26}	-0.54	-0.41
R_{27}	-0.42	0.36
R_{28}	-3.00	-0.58
R_{29}	-1.59	-0.58
R_{30}	-0.05	0.18
R_{31}	-0.10	0.06
R_{32}	0.11	0.29
R_{33}	-0.38	-0.08
R_{34}	-0.51	-0.14
R_{35}	0.00	0.04
R_{36}	0.03	0.07
R_{37}	0.39	0.67
R_{38}	0.34	0.41
R_{39}	0.09	0.38
R_{40}	0.58	3.00
R_{41}	0.58	1.65
R_{42}	0.04	0.29
R_{43}	0.58	1.17
R_{44}	-0.58	0.05
R_{45}	-0.27	0.06

ID	Measured changes in fluxes: $\Delta\log_2 J$	
	minimum	maximum
R_{48}	-0.49	-0.26
R_{49}	-0.53	-0.18
R_{50}	0.58	3.00
R_{51}	-0.21	0.19
R_{52}	-0.64	0.93
R_{53}	-0.93	0.07
R_{54}	-0.72	-0.16
R_{55}	-0.45	-0.21
R_{56}	-0.65	-0.48
R_{57}	-3.00	-0.58
R_{58}	-0.01	0.10
R_{59}	0.58	1.09
R_{60}	0.33	0.48
R_{61}	0.58	3.00
R_{62}	0.09	0.68
R_{63}	0.58	1.29
R_{64}	0.58	3.00
R_{65}	-0.48	-0.04
R_{66}	0.58	1.88
R_{67}	0.58	3.00
R_{68}	-1.56	0.78
R_{69}	0.22	0.39
R_{70}	0.14	0.44
R_{71}	-1.01	2.34
R_{72}	-0.36	1.32
R_{73}	0.58	3.00
R_{74}	-0.12	-0.08
R_{75}	0.19	0.47
R_{76}	-1.23	-0.44

ID	Me. changes in concentrations: $\Delta\log_2 x$	
	minimum	maximum
Ala	-0.08	0.09
Asp	0.58	1.34
Cit	0.12	0.15
Glu	0.54	0.62
αKG	0.19	0.37
Mal	0.56	0.72
NADPH	-0.39	-0.12
Pyr	0.07	0.58

ID	Meas. changes in enz. activities: $\Delta\log_2 v$	
	minimum	maximum
R_{39}	$\min \Delta\log_2 J_{39}$	$\max \Delta\log_2 J_{39}$

R_{46}	-0.24	-0.06	⋮	⋮	⋮
R_{47}	-0.26	0.21	R_{76}	$\min \Delta \log_2 J_{76}$	$\max \Delta \log_2 J_{76}$

Higher minimum: $\log_2 1.5 = 0.58$; lower maximum: $-\log_2 1.5 = -0.58$.
