# Supplementary material for: Integrating systemic and molecular levels to infer key drivers sustaining metabolic adaptations
Source: PLoS Comput Biol. 2021 Jul 23;17(7):e1009234. doi: 10.1371/journal.pcbi.1009234 (PMC8336858; doi:10.1371/journal.pcbi.1009234)
Supplement: Attachment — Submitted filename: Response to reviewers.docx [file pcbi.1009234.s004.docx]

Dear Editors, Dr. Kiran Raosaheb Patil and Dr. Feilim Mac Gabhann,

We thank the reviewers for their valuable suggestions and for raising important issues. In consideration of these comments, we revised the manuscript as follows:

**Responses to the Editor’s comments:**

In order to give particular attention to the data availability and providing a more accurate presentation on novelty and general applicability, we have introduced the following changes:

- Data availability. A detailed description of the procedures and data used in both the glycolysis-case study and the cancer-case study has been rewritten in the Material and methods section, describing them point by point and following referee’s suggestions. In order to improve access to these data, appropriate links to these descriptions have been added in the main text.
- Novelty. We agree with the concern of the editors and reviewers that in some parts of the Results section it is difficult to distinguish which pieces are novel work and which are already known. For a more accurate presentation of novelty, entire sections discussing what is already known have been moved to the Introduction and have been rewritten.
- General applicability. To ensure complete understanding and reproducibility:
  - We have eliminated the formulation done using response coefficients and special elasticities, presenting the formulation for large changes from the beginning and for the toy model.
  - We have edited old Fig 1, which presented a part of the analysis that is not new, and converted it in Fig 2. To be consistent with the network scheme for the glycolysis-case presented in this new Fig 2, a new figure (Fig 3) has been created with a network scheme for the cancer-case study, which indicates the genes downregulated and upregulated that are analyzed.
  - A new section describing the flow chart of the proposed method has been created. This flow chart is associated with a new version of Fig 2, which has been edited as a flow chart and renamed as Fig 1.
  - Old Fig 2 and old Fig 4, renamed as Fig 1 and Fig 5, have been edited to describe the domains using the inequality notation to be consistent with the inequality notation used in the equations.
  - Old Fig 3 has been renamed as Fig 4.
  - S3 Table, which was supporting the sampling strategy for metabolite elasticities, has been eliminated and substituted by a detailed explanation in the Material and methods section.
  - S4 Table has been renamed as S3 Table
  - Finally, a new document is provided with the instructions for use of the mathematical notebook provided to solve final domains and identify negative and positive signs starting from calculated control coefficients and initial values. These files are freely available for download at Mendeley Data (<http://dx.doi.org/10.17632/9wyzpjzwkx.1>). [Restricted access during the submission period: https://data.mendeley.com/datasets/9wyzpjzwkx/draft?a=7ce0d31a-6d9c-4544-9aa3-e43697f27c38]

Finally, other major and minor changes have been introduced in response to referee’s suggestions.

**Responses to Reviewer #1:**

*Overview: This manuscript presents a method to identify metabolites and enzyme-catalyzed reactions that significantly influence the metabolic response (reprogramming) to perturbations. The approach is applied for a simple model with detailed rate expressions, as well as a larger model. The method put forth in this paper is useful and relevant. However, details are missing the prevent a full understanding of the approach and its applicability.*

**Specific comments:**

*(1) Details regarding implementation of the approach are missing: how were the bounds of the control coefficients for sampling set? Is a set of 100 ensemble formulated problems sufficient? How are the fluxes determined for the larger model?*

- Regarding the control coefficient bounds, a detailed explanation is provided in lines 859-862 of the revised manuscript: “*Only matrices with all control coefficients within a -3.5 to +3.5 range were selected.* *As for the differences in concentrations, fluxes and individual activities, values outside this enclosure were not accepted. Although it is arbitrarily set, this enclosure was added to avoid models with unrealistic sensitivities.*”
- Regarding the ensemble of formulated problems, we repeated our analysis up to 100 times, which was enough to see when the same change trends are consistently repeated. This number could be adapted depending on the size of the model. Our aim was not to establish a test with statistical value to determine the level of significance of the repetitions. Such an objective could be addressed in future work. A sentence has been added in the Discussion (lines 641-644 of the revised manuscript) addressing this issue: “*The set of 100 ensemble formulated problems was sufficient to provide a qualitative assessment of the significance of the changes. However, this number should be adapted depending on the size of the analyzed problem, and a more robust test with statistical value could be addressed in future work.*”
- Regarding the determination of the fluxes for the larger model, the case study was based on our previously published work covering the effects of CDK4/6 inhibition in the HCT116 colon cancer cell line [29]. The fluxes were estimated in this published paper. A detailed explanation is provided in the Material and methods section and has been in part rewritten (lines 684-702 in the revised manuscript); “*A second study spanning all central carbon metabolism was also analyzed, based on our previous work covering the effects of CDK4/6 inhibition in the HCT116 colon cancer cell line [50]. The study characterized the metabolic reprogramming, both at the systemic and molecular levels, in response to CDK4/6 inhibition. To this aim, at the molecular level, downregulations and upregulations for gene levels were measured using transcriptome microarrays and qRT-PCR. At the systemic level, ~~changed~~ differences on the levels for all fluxes and some metabolites (alanine, aspartate, citrate, glutamate, malate, NADPH, pyruvate, and α-ketoglutarate) were measured for control cells (before perturbation) and for CDK4/6-inhibited cells. A stoichiometric model for the central carbon metabolism was constructed and solved by applying SIRM techniques to estimate all flux distributions throughout the metabolic network, including forward and reverse reaction rates when required. For this, direct extracellular measurements, such as oxygen consumption, and consumption and production rates for glucose, lactate, and all amino acids, as well as protein synthesis rates, were combined with 13C isotopologue (mass isotopomer) enrichments measured in several metabolites. Such enrichments emerge from the propagation of 13C from labeled glucose and glutamine to metabolites through the network and are informative of the underlying flux distribution. The metabolites analyzed for label propagation included lactate and amino acids from incubation media, glycogen, ribose from RNA, palmitate, and several other internal metabolites.*”.

*(2) For some of the predicted metabolic activities shown in Table 2 and compared to experimental data, less than the majority of the set of 100 solutions have only positive (or negative) fluxes. Can conclusions truly be drawn from this, without a consensus? Do the authors expect that a certain percentage is needed to make a clear conclusion?*

- As an example, to illustrate the utility of the proposed procedure, we evaluated if our analysis in the cancer-case study supported a possible role for the measured changes in gene expression as drivers of the metabolic adaptations. To qualitatively assess it, we used the percentages of negative and positive signs. We have added the following sentences in the main text (lines 575-585 of the revised manuscript) to provide a better description of the comparison of predicted and measured changes: “*The percentages of negative and positive signs were used to assess if the computational analysis qualitatively supports for the measured changes in gene expression a possible role for them as molecular drivers of the metabolic adaptations. There was in general a good correspondence that supported this~~e~~ role ~~of~~ for ~~a part~~most of these analyzed genes ~~as potential drivers sustaining the metabolic adaptation~~. In Table 2 (last column) we have classified these changes as “supported” or “supported, but sampling dependent” and “not supported, but sampling dependent”. The role as key drivers of the metabolic adaptation for IDH2 and GLS1 was supported by the high percentages of signs indicating that the changes predicted in metabolic activities are going in the same direction than the changes measured in gene expression. For the rest, the lower percentages do not allow for any final conclusion, rather than this will depend on the sampling of metabolite elasticities*.”

*(3) The paper describes one* *contradiction between the predicted metabolic activities and experimental data (PDH). Were there other discrepancies between the model and data? These should be described for full disclosure, as it helps the reader place the predictions and the approach in context.*

- In our comparison with the gene expression data, the contradiction between the predicted metabolic activities and gene expression experimental data for PDH was the only discrepancy found. Anyway, this type of discrepancy is useful as it indicates a weakness in our analysis, suggesting that additional information is required to explain the observed behavior. We have added the following sentence to emphasize the value of this contradiction in the proposed analysis (lines 592-596 in the revised manuscript): “*Although some negative signs were predicted for the HIF1-dependent mitochondrial PDH (pyruvate dehydrogenase complex) activity, the downregulation of PDK1/PDK3 should drive an increase in PDH activity.* *This discrepancy is helpful as it indicates that additional information for post-translationally regulated enzymes is required to explain the observed behavior.*”

*(4) What does the last column in Table 2 mean?*

- As commented above (point 2) an example of applying the proposed procedure, we evaluated if our analysis supported a possible role for the measured changes in gene expression as drivers of the metabolic adaptations. Combined with the sentence added (see discussion in point 2), we have modified the last column in table 2 to better explain the meaning of this last column:

| **Gene ID** | **qRT-PCR** | **Affymetrix GeneChips** | **Affected ind. activity ID** | **% -** | **% +** | **Role as a driver of the metabolic adapt.** |
| --- | --- | --- | --- | --- | --- | --- |
| *SLC2A3* | -0.8 | -1.2 | Glc-transp/HK/ PFK/PGM/ENO (HIF1) | 40 | 0 | supported, but sampling dependent |
| *HK2* | -0.9 | -0.7 |  |  |  |  |
| *PFKFB4* | -1.8 | -0.8 |  |  |  |  |
| *ENO2* | -2.3 | -1.3 |  |  |  |  |
| *PDK1* | -1.2 | -0.5 | PDH | 36 | 0 | not supported, but sampling dependent |
| *PDK3* | -1.2 | -1.3 |  |  |  |  |
| *IDH2* | -0.7 | -0.6 | ACO/IDH | 93 | 0 | supported |
| *GLS1* | +1.0 | +0.6 | GLS | 0 | 87 | supported |

*(5) It is not clear how some constraints were established. For example, in the colon cancer model example, an assumption about* *coordinated regulation of a block of enzymes was made. How was this determined? What would the results be if that assumption was relaxed – would the results compare to experiments? Other places where additional constraints are applied but not explained: lines 305-307, red font in Fig. 2, Table S4.*

- The constraints in lines 305-307, the red font in Fig2 and Table S4, refer to the initial and final domains of possible values for the variables (differences in flux reactions, metabolite concentration, and individual activities) used in the glycolysis-case and the cancer-case studies, respectively. As discussed above, in the revised version, a new section has been created to describe the whole procedure, which includes an explanation (lines 347-361 in the revised version) explaining how these domains and the values of their bounds were established: “*The variables are continuous domains measuring the differences in reaction fluxes, metabolite concentrations, and individual activities. First, common lower and upper bounds are set for the domains of all variables to be enclosed between a minimum lower bound and a maximum upper bound, assuming that differences outside this enclosure are not accepted. Although this enclosure is arbitrarily set, it can be adapted depending on the observed changes and contributes to reducing the space of solutions. In both case studies (initial domains in panel C in Fig 1 for glycolysis-case), common lower and upper bounds were set to be -3 and +3, which was consistent with the magnitude of expected changes. Second, the domains for differences in concentrations, fluxes, and individual activities measured experimentally are additionally restricted using the measured confidence intervals as bounds. These additional restrictions are a fundamental part of the problem formulation because the objective is to see the effect of these additional restrictions on all other variables, to finally see if new previously unknown sign-fixed domains appear.*”.
- Regarding the coordinated regulation of a block of enzymes, a paragraph has been added explaining how this assumption was established (lines 548-557 in the revised version): “*A subset of them was experimentally verified by qRT-PCR (Fig 3). Also, our experimental observations supported previous reports sustaining that part of these differentially expressed genes are HIF1-dependent genes [69-71]. The effect on gene expression of hypoxia and CDK4/6 inhibition presented the opposite direction for several genes [50]. In particular, among the selected genes, SLC2A3, HK2, PFKFB4, ENO2, PDK1 and PDK3 were upregulated in response to hypoxia and downregulated in response to CDK4/6 inhibition. Based on these evidences, we assumed a coordinated regulation by the transcriptional regulator HIF1 of glycolytic SLC2A3, HK2, PFKFB4, and ENO2 genes, and therefore a coordinated regulation of the individual activities of their encoded products (Glc transp, HK, PFK, and ENO, respectively) (equation (14)).*”. Also, we have rewritten the paragraph discussing what happens if this assumption is eliminated (lines 588-591 in the revised manuscript): “*This assumption strongly reduced the space of solutions, which facilitate the appearance of signs supporting the requirement of such transcriptional co-regulation, as measured experimentally. However, the signs (in orange, Fig 4) disappeared when the constraints assuming the coordinated regulation were not included.*”.

*(6) Is the black “-“ sign for the HK reaction in the 3rd model formulation a typo? Should this be orange/red?*

- No, it is not a typo; this indicates that in the 3^rd^ model formulation, the sampled elasticities are enough to constraint the individual activity of hexokinase (HK) to decrease, while for the rest of the model formulations are required the additional constraints connecting the changes in the activity of HK (*R_02_*) with those for glucose transport (Glc transp) (*R_01_*), phosphofructokinase (PFK) (*R_04_*) and enolase (ENO) (*R_08_*): ($\Delta\log v_{01}=\Delta\log v_{02}=\Delta\log v_{04}=\Delta\log v_{08}$). A sentence has been added describing this particular case (lines 520-524 in the revised manuscript): “*The constraints associated with the sampled elasticities can be enough to require a decrease in the individual activity of HK, as shown in the 3rd model formulation (Fig 4), while for the rest of the model formulations the additional constraints in equation (14) connecting the changes in the activity of HK with those for Glc transp, PFK and ENO are needed*”

*(7) The authors have not explained what the union and intersection for the final domains mean.*

- The analysis of the unions and intersections provides complementary information to that provided by the signs. The following sentence has been added to explain the meaning of these unions and intersections (lines 489-496 in the revised manuscript): “*The analysis of the unions and intersections in Fig 5 provides complementary information to that provided by the signs. Although the applied analysis has a qualitative value, such unions and intersections provide a numerical summary to assess the magnitude of these changes, which was significant in all the cases, and also (as the signs) tell us about the dependence on the sampling. A coincidence of the lower and upper bounds of the unions and the intersections will correspond to a no dependence on the sampling of control coefficients.*”.

*(8) In the current form, it is just not clear how one could implement the approach, especially considering point 5 above. The authors can clarify how to apply their method.*

- As discussed above, to ensure a complete understanding and reproducibility, together with the various improvements suggested by the referees, a flow chart of the proposed method has been added in the main text. This flow chart is accompanied by a detailed description of the assumptions and data applied in both case studies provided in the Material and methods section and the supplementary material, which have been conveniently rewritten. Finally, the instructions to use the Mathematica notebook have been improved. We hope that these changes are deemed appropriate by the referee to have a complete understanding of the approach and its applicability.

**Responses to Reviewer #2:**

*The authors present a mathematical framework that combines metabolic control analysis with linear programming to estimate ranges of response coefficients in kinetic models with incomplete information. Although the work looks interesting, I find the structure of the manuscript is very hard to follow and* *I could not fully understand what are the advantages of the proposed method and how they differ from previous work. Please find some more detailed comments bellow.*

*- The results section begins by introducing basic definitions of metabolic control analysis (control coefficients, elasticities, summation and connectivity theorems). I don't understand why this is all introduced in the results section.*

- As commented above, for a more accurate distinction between what is already known and novelty, we have moved the basic definitions of the metabolic control analysis section and Table 1 to the Introduction (lines 104-134 in the revised manuscript).

*- The authors then show how the basic theorems of MCA can be re-written in a different structure that creates a linear system of equations. Throughout this section the authors often mention how different MCA and BST systems (GMA, S-Systems, lin-log) have different advantages. Again, I don't see why this is included in the results and makes it hard to understand which pieces of what is being presented is novel work and what is already known.*

- As for the basic definitions of metabolic control analysis, we have moved this section to the Introduction (lines 136-182 in the revised manuscript).

*- The toy case study did not help to understand the method. For instance, Figure 1 shows that the control coefficients are derived from the ODE model, but does not explain how.*

- Regarding the toy model, we have changed the way that the toy case-study is presented, in order to make it more useful to understand the whole method (see the response for the next comment).
- Regarding the derivation of the control coefficients, an explanation has been added in the Material and methods section (lines 673-681 in the revised manuscript): “*A unique set of control coefficients was derived from the detailed kinetic model, therefore permitting a unique problem formulation. Control coefficients were estimated by following this procedure: 1) steady-state concentrations and fluxes are calculated by setting the initial conditions and solving the ODE; 2) metabolite elasticities are calculated using the steady state concentrations and fluxes; 3) stoichiometric flux and concentration dependencies are derived and used in the form of flux and concentration ratios, together with metabolite elasticities, to calculate control coefficients by applying the matrix method developed by Cascante et al. [31, 32] (panel D in Fig 2)*”.

*- When presenting the larger case study, called "Response to a large perturbation", the authors reformulate the previously introduced linear system, with a slightly different one that replaces the response coefficient with logarithms, under the justification that the one used in the first case study is only valid for infinitesimal perturbations, which not appropriate for real case studies. So why not present this formulation from the beginning?*

- Although the toy model is useful as a tool to ensure a complete understanding and applicability of the proposed analysis, we agree with the referee about the lower utility of the maximization/minimization LP formulation performed in the context of infinitesimal changes, response coefficients, and special elasticities. Therefore, the description of the whole procedure is uselessly complex. Accordingly, following the referee’s suggestion, we have eliminated the formulation done using response coefficients and special elasticities, presenting the formulation for large changes from the beginning and for the toy model.

*- The final results of the cancer case study do seem relevant, and they are supported by the authors own experimental work, but* *it is very hard to access their relevance more clearly without a better understanding of the mathematical framework.*

- We thank the reviewer for his comments and hope that the modifications introduced in the revised version of the paper provide a better understanding of the mathematical framework, the advantages of the proposed method, and how they differ from previous work.

**Responses to Reviewer #3:**

*The manuscript describes a computational methodology based on combining metabolic control analysis with linear programming to identify major metabolic adaptions to small and large perturbations, such as those induced by drug therapy. As such, the work is thematically relevant to the scope of the journal. The overall idea is worthy of publication, however there are a number of concerns and questions that need to be addressed in order for the manuscript to be publishable.*

*A major concern comes from the authors’ claim that the methodology presented can address system responses to large perturbations. However, the whole premise of the work is based on linearization around some state(s), which by default is valid for small perturbations around those states. The validity of linearization of a very complex system when applying large perturbation is at the least questionable and needs to be properly justified.*

*Another major concern has to do with the novelty of the work presented. Performing linear optimization for metabolic control analysis (MCA) is certainly not novel, as also evidenced by the various references cited in the manuscript. Discussion of the novelty of the work, therefore needs to be significantly improved.*

*Also the mathematical discussion needs to be tightened up and sorted out in places.*

**Detailed points and questions are listed below to aid the authors in preparing an improved manuscript:**

*1. In the beginning of the results section (lines 150-152, the authors state that in this manuscript “MCA has been defined using slightly different formulations, which are also related with tis used in BST”. This phrase in addition to being syntactically wrong does not clarify the novelty of the work. In fact a large portion of the results section intermittently combines background information with seemingly new information, to the point that any potential novelty is hidden. I would recommend a rewrite, where the background is clearly discernible from the actual results.*

- As commented above, for a more accurate distinction between what is already known and novelty, entire parts of the Results section discussing what is already known have been rewritten and moved to the Introduction. The sentence has been rewritten (lines 107-109 in the revised manuscript) as “*MCA has been defined using formulations that are slightly different ~~MCA has been defined using slightly different formulations~~ [15, 16, 18, 19, 28], although equivalent, to those used in BST ~~which are also related with this used in BST~~ [21, 29]*”.

*2. Table 1 give definitions of sensitivity coefficients as used in the manuscript. A. It is not clear why dx_i/dNu_k is not a partial differential. In the same way dNu_k/dp should NOT be a partial differential. B. It is not clear which of these deifinitions are new as in one or another form they have been used before. This needs to be clarified as it it forms the basis of the work presented.*

A. Regarding the use of total derivatives, it is common to define response coefficients, control coefficients, and elasticities using partial derivatives. However, some authors use total derivatives to describe the response and control coefficients, while partial derivatives are used for elasticities. This helps to emphasize that for response and control coefficients, the entire system is allowed to relax to a new steady-state after the perturbation in the enzyme activity, while for elasticities, the reactions are isolated from the rest of the system. To clarify this point, the following sentence has been modified in the legend of Table 1: “*In control and response coefficient, the variations in systemic concentrations and fluxes are the result of the collective adaptation of the entire system after a transient period of adjustment**, which is indicated using total derivatives [19]. In contrast, regarding elasticities, the changes in transport or enzyme activities happen as isolated individual processes.*”.
B. As part of the improved distinction between what is already known and novelty, to avoid any confusion, we have moved the basic definitions of metabolic control analysis section and Table 1 to the Introduction (lines 104-152 in the revised manuscript).

*3. Line 184: It is clearly the chain rule and not chain’s rule applied here.*

- “Chain’s rule” has been appropriately corrected to “Chain rule” (line 140 in the revised manuscript).

*4. Equations 3 and 4 are derived by applying the chain rule on equations 1 and 2: A. The novelty (or not) of this formulation needs to be better discussed as seemingly references 31-34 us ethe same premise. B. It is not clear how these equations imply some kind of two level hierarchy. Response coefficients are direct functions of control coefficients, but once these are somehow calculated or estimated, then response coefficients are also known (fully or partially), so the two-level approach implied needs to be better thought or explained.*

A. As a part of the improved distinction between what is already known and novelty, the application of the chain rule and the associated references have been moved from the Results section to the Introduction (lines 136-152 in the revised manuscript).
B. The distinction of the two levels of hierarchy is fundamental to understand the philosophy under our analysis. The text has been rewritten to clarify the meaning of this two-level-of-hierarchy (lines 259-269 in the revised manuscript): “*Given a mathematical model that constrains the values of variables, such as concentrations, fluxes, and individual activities, this model must allow for the description of the different experimental situations that may potentially occur. Once the model is defined, it can be applied by adjusting the variables to reproduce particular conditions. In MCA, in the context of equations (3) and (4), or equations (6) and (7) for larger changes, the first level of “model description” can be provided by using control coefficients with fixed values. Once the model is established, a second level of “model application” can be done by adjusting the fluxes, concentrations, and individual activities. These variables can be taken as continuous domains in the form of bounded (closed) intervals measuring the differences in reaction fluxes, metabolite concentrations, and individual activities that are comparing the values before and after the adaptation to a metabolic perturbation.”*

*5. The “**inference by bound contraction section is written pretty much as a literature review section and not particularly as a results one, so this needs to be seriously addressed.*

- As a part of the improved distinction between what is already known and novelty, we have moved the literature review part of the “inference by bound contraction” section to the Introduction (lines 153-182 in the revised manuscript).

*6. Line 233-234: What are control coefficients fixed to? This is normally the most difficult information to obtain for a network*

- Following the referee’s #2 suggestion, we have eliminated the formulation done using response coefficients and special elasticities, presenting the formulation for large changes from the beginning and for the toy model. However, as discussed in the answer for question 4, for large changes in the context of MCA and our proposed analysis, the known values of the control coefficients represent the first level of “model description”. Control coefficients are fixed according to the available information. For clarity, we have rewritten the sentence stated in line 233-234 (old version) (lines 275-278 the revised version): “*In this reformulation, differences in fluxes, concentrations, and individual activities ~~response coefficients and special elasticities~~ can be taken as decision variables in linear constraints, with ~~fixed~~ control coefficients taken as the constant coefficients**, fixed according to the available information.*”

*7. Line 248: In which cases is the problem not solvable? How is this assessed and/or addressed?*

- The problem is solvable if all the model constraints and initial domains are satisfied. We can state that all initial constraints are compatible. This has been the situation for the unique formulation problem in the glycolysis-case study, but not for 16 of the 100 formulated problems in the cancer-case study, which were not compatible with all initial domains and constraints. The 16 problems that were not satisfied were discarded. For clarity, we have accompanied the sentence stated in line 248 (old version) with additional sentences (lines 292-296 of the revised version): “*The problem is solvable if all the model constraints and initial domains are satisfied, in other words, if all initial constraints are compatible. If the problem is solvable, each initial domain should contain at least one value satisfying all inequalities and constraints in (11).* *Otherwise, the problem is discarded.*”

*8. The authors are essentially “inverting” flux variability analysis procedure by fixing fluxes and calculating the min-max of response coefficients. This is a nice approach. It needs however to be clarified what is the final domain reduced from (as stated in line 258-259), i.e. what is the initial domain?*

- As discussed above, following the referee’s #2 suggestion, we have eliminated the formulation done using response coefficients and special elasticities, presenting the formulation for large changes from the beginning and for the toy model. However, the question remains for large changes. As discussed in the response to question 5 of Reviewer #1, a new section has been created to describe the whole procedure in the revised version, including an explanation (lines 347-361 in the revised version) of how initial and final domains and the values of their bounds were established: “*The variables are continuous domains measuring the differences in reaction fluxes, metabolite concentrations, and individual activities. First, common lower and upper bounds are set for the domains of all variables to be enclosed between a minimum lower bound and a maximum upper bound, assuming that differences outside this enclosure are not accepted. Although this enclosure is arbitrarily set, it can be adapted depending on the observed changes and contributes to reducing the space of solutions. In both case studies (initial domains in panel C in Fig 1 for glycolysis-case), common lower and upper bounds were set to be -3 and +3, which was consistent with the magnitude of expected changes. Second, the domains for differences in concentrations, fluxes, and individual activities measured experimentally are additionally restricted using the measured confidence intervals as bounds. These additional restrictions are a fundamental part of the problem formulation because the objective is to see the effect of these additional restrictions on all other variables, to finally see if new previously unknown sign-fixed domains appear.*”. For clarity, we have rewritten the sentence stated in line 258-259 (old version) (lines 304-306 the revised version): “*Starting with an initial domain, t~~T~~aking the minimum and maximum solutions, this LP formulation provides a reduced final domain ~~(reduced continuous subdomain of the initial domain)~~ for each decision variable.*”

*9. What is the reduced domain for the response coefficients in line 305-307?*

- Please, see the response to question 8.

*10. Regarding the response to large perturbations section: The main question is how are equations 7-12 applicable as they are essentially linearizations? What makes this a valid approach for a complex highly nonlinear system? Linearisation for small perturbations is a logical approach. Applying this to large perturbations is a logical leap.*

- We thank the reviewer for raising this important issue. Our objective was qualitative, but not quantitative. We are interested in capturing the trends of the changes, increases or decreases, and if these trends are significant. Accordingly, our objective is to obtain positive and negative trends of change expressed as positive and negative signs. Sentences have been included in the Introduction and Discussion raising this objective:
- Added to the Introduction (lines 319-324 in the revised manuscript): “*As the problem formulation is based on linearization, our objective can be qualitative, but not quantitative. Accordingly, the objective was to obtain collections of negative and positive signs, looking to capture the trends of the changes, decreases (negative) or increases (positive), and whether these trends are significant. ~~Looking for required d~~Sign-fixed final domains ~~ecreases or increases~~that are required to explain all the associated constraints will be identified from~~, our objective was to use these ranges of variability to identify sign-fixed final~~ domains that have only negative or only positive values~~, respectively”~~.*
- Added to the Discussion (lines 619-628 in the revised manuscript): “*With the goal of identifying molecular drivers of changes observed in metabolic adaptations to perturbations, the applied analysis is based on a combination of the MCA description of response coefficients as a function of special elasticities and control coefficients [30-33], and the subsequent reformulation as part of a linear optimization-based strategy for bound contraction. This reformulation takes advantage of linearization around a reference steady-state and therefore cannot be applied for large changes with a quantitative aim. However, it can be applied to capture the trends of the changes, increases or decreases. Accordingly, the objective was to obtain a collection of positive and negative signs, whose repetition will allow us to assess the significance of these changes when the problem is coupled with uncertainty*.”

*11. Taking into account the complexity of the second case study (as the glycolysis one is essentially used as a validation problem) how is it expected to know flux control coefficients and in addition those to be constants? In my view this is the trickiest information to obtain. If these are obtained by flux control analysis using constant response coefficients, how is this not undermining the information obtained in this work?*

- In the glycolysis-case study a unique matrix of control coefficients was derived from a detailed kinetic model built around a well-defined steady-state. Accordingly, a unique problem formulation is done. In contrast, for the cancer-case, coupled with uncertainty, multiple sets of control coefficients were estimated, therefore giving multiple problem formulations. In this second case study, a more global perspective is applied, as explained in the following paragraph that has been in partially rewritten (line 430-443 in the revised version): “*In the glycolysis-case study, following a local perspective, a unique matrix of control coefficients was derived from a detailed kinetic model built around a well-defined steady-state. Therefore, by solving a unique set of linear constraints. In contrast, the cancer-case study was done under realistic experimental conditions, involving a complex metabolic response to CDK4/6 inhibition, with two different steady states, before and after CDK4/6 inhibition. On the one hand, a unique matrix of known and constant control coefficients cannot adequately be applied because the values change during the adaptation to the large perturbation. On the other hand, there is limited availability of data to estimate metabolite elasticities and the ratios among stoichiometrically dependent fluxes and concentrations. Moving from the local analysis, both limitations can be tackled simultaneously by applying a more global analysis, solving ensembles of the complete proposed problem, each one associated with a matrix of control coefficients generated by random sampling methods, therefore covering a wider parameter space as described by Kent et al. [65].*”

*12. Line 370: Why is the initial domain set between -3 and 3? Is it just heuristics?*

- Please, see the response to question 8.

*13. Line 437: how was reversibility (or not) decided and fixed for each of the reactions involved? This needs to be clarified in the manuscript.*

In the absence of data for a clear description of each reaction's level of reversibility, this was decided, first, by applying our general knowledge to select the more irreversible processes. For boundary processes involving transport and simplified pools involving several reactions, or processes known to be far to the equilibrium in physiological conditions, we assumed irreversibility. Second, for processes known to be more reversible, when possible, from measured reverse to forward ratios, taking advantage of the measured forward and reverse rates that we obtained in the experiments of ^13^C propagation in the paper we previously published. However, this can be applied just as an approximation because the experiments were not designed with this purpose. Accordingly, we have rewritten the description for the selection of the values of the displacements from equilibrium (lines 824-831 in the revised manuscript): “*This required an estimation of the disequilibrium ratios (S2 Table). Most of the reactions associated with transport and simplified pools of reactions accounting for boundary processes were assumed to be far from equilibrium. For some other processes, disequilibrium ratios known to be closer to equilibrium in biological conditions were set to ρ = 0.9, except for some cases for which ρ were corrected to lower values ~~from~~ using as an approximation ~~the~~ SIRM-estimated reverse to forward ~~and reverse~~ rates (see equation (19)). The upper level of 0.9 for ρ was selected as a compromise to avoid unrealistic values of control coefficients*.”

*14. In line 481, which is the original paper mentioned?*

- The original paper corresponds to our previous work covering the effects of CDK4/6 inhibition in the HCT116 colon cancer cell line. Several references have been added, and the text has been rewritten (lines 549-554 in the revised manuscript): “*Also, our experimental observations supported previous reports sustaining that part of these differentially expressed genes are HIF1-dependent genes [69-71]. The effect on gene expression of hypoxia and CDK4/6 inhibition was the opposite for several genes [50]. In particular, among the selected genes, SLC2A3, HK2, PFKFB4, ENO2, PDK1 and PDK3 were upregulated in response to hypoxia and downregulated in response to CDK4/6 inhibition*.”.

*15. The “discussion” section explains a number of things that remain vague in the results section. So as mentioned above a significant rewrite need to take place.*

- As discussed above, together with the various improvements suggested by the referee’s, a large set of changes have been introduced to ensure complete understanding and reproducibility. We have taken special care in making these changes, ensuring that there is total correspondence with what is discussed in the Discussion section. We hope that these changes are deemed appropriate by the referee to have a complete understanding of the approach and its applicability.

*16. Lin 731. It is not clear to what equations 20-22 are integrated. Which are the limits etc. This needs to be clearly discussed in the manuscript.*

- In order to clarify the integration of equations 20-22, a sentence has been rewritten (lines 808-811 in the revised manuscript) as “*Taking* $0<\rho<1$*, and therefore for* $v>0$*, by substituting in equation (20)* $ɛ_{S}^{vr}$ *and* $ɛ_{S}^{vf}$*according to the equality in equation (21) for S (*$ɛ_{R}^{vr}=ɛ_{R}^{vf}-1 and ɛ_{R}^{vf}=ɛ_{R}^{vr}+1$*) and* $ɛ_{P}^{vr}$ *and* $ɛ_{P}^{vf}$ *according to the equality in equation (22) for P (*$ɛ_{R}^{vr}=ɛ_{R}^{vf}+1 and ɛ_{R}^{vf}=ɛ_{R}^{vr}-1$*), the following equations are derived ~~Integrating equations (20) and (21,22)~~ [15,79], ~~and assuming that~~* $v>0$ *~~(and then~~* $0<\rho<1$*~~), the following equations are derived~~ respectively:*”.
