# Supplementary material for: Integrating systemic and molecular levels to infer key drivers sustaining metabolic adaptations
Source: PLoS Comput Biol. 2021 Jul 23;17(7):e1009234. doi: 10.1371/journal.pcbi.1009234 (PMC8336858; doi:10.1371/journal.pcbi.1009234)
Supplement: Attachment — Submitted filename: Response to reviewers.docx [file pcbi.1009234.s005.docx]

Dear Editors, Dr. Kiran Raosaheb Patil and Dr. Feilim Mac Gabhann,

We want to thank the reviewers again for their positive and constructive comments. In consideration of these comments, we revised the manuscript as follows:

**Responses to the Editor’s comments:**

In order to make all computational code underlying the findings in their manuscript fully available, an additional Mathematica notebook is provided to generate the control coefficients by the sampling of metabolite elasticities for the cancer-case study. This file, together with associated excel files with data and a document with instructions for the use of the mathematical notebook, are freely available for download at Mendeley Data (<http://dx.doi.org/10.17632/9wyzpjzwkx.1>). [Restricted access during the submission period: https://data.mendeley.com/datasets/9wyzpjzwkx/draft?a=7ce0d31a-6d9c-4544-9aa3-e43697f27c38]

Finally, other minor changes have been introduced in response to the referee’s suggestions and we have carefully revised the manuscript.

**Responses to Reviewer #2:**

*The authors have addressed all my comments, which were mainly concerning the structure of the manuscript. The revised version has substantially improved with regard to readability.*

*I have only a few additional minor suggestions:*

*- Figure 2 (network diagram) should come before Figure 1 (matrix coefficients of the network).*

- Figures 1 and 2 have been re-labeled (Figure 1 is the old Figure 2, and Figure 2 is the old Figure 1). So, following the reviewer’s suggestion, in the revised version, the old Figure 2 comes before the old Figure 1.

*- The percentage gain metric should be explained somewhere, preferably before its first referral in figure 1.*

A sentence has been added describing the percentage gain metric (lines 378-380 in the revised manuscript with changes highlighted): “As a measure of this bound contraction, a percentage gain metric has been added for each variable to quantify the percentage of reduction in the size of their final domains with respect to their initial domains.”

*- Panels B and C in figure 1 seem to be incorrectly labeled.*

- We thank the reviewer for his careful revision. Unfortunately, panels B and C in the old Figure 1 were incorrectly labeled. In the revised version, panel B is the old panel C, and panel C is the old panel B.
